# Cavernous Sinus Thrombosis related to Orbital Cellulitis Serious Complication to Prevent: a case report and literature review

**DOI:** 10.1016/j.amsu.2021.01.036

**Published:** 2021-01-18

**Authors:** Rachid Aloua, Ouassime Kerdoud, Faiçal Slimani

**Affiliations:** a Faculty of Medicine and Pharmacy, Hassan II University of Casablanca, B.P 5696, Casablanca, Morocco; b Oral and Maxillofacial Surgery Department, CHU Ibn Rochd, B.P 2698, Casablanca, Morocco

**Keywords:** Orbital cellulitis, Cavernous sinus thrombosis, Complication, Prognosis

## Abstract

**Introduction:**

The authors report a case which aims to underline the importance of multidisciplinary management and rapid diagnosis of orbital cellulitis, for an adequate treatment of ocular damages and related complications, to prevent serious and permanent sequelae and avoid a fatal prognosis.

**Presentation of case:**

A 61-year-old female reported to the oral and maxillofacial surgery department after she was dragged around for two months. She presented with a right facial swelling and orbital apex syndrome including proptosis, ophthalmoplegia and ptosis.

**Discussion:**

Complications of orbital cellulitis may be limited to the orbit, such as subperiosteal or orbital abscess, optic neuritis, blindness, or intracranial such as meningitis, sinus cavernous thrombosis, cerebral abscess and even death.

**Conclusion:**

Maxillofacial surgeons must be aware of this complication in a multidisciplinary context to adopt adequate treatment as soon as possible.

## Highlights

• Orbital cellulitis is a rare ophthalmic disease due to pansinusitis with hard maxillofacial management.
• Cavernous sinus thrombosis is a rare but highly fatal complication of orbital cellulitis.
• Early and appropriate treatment can improve the bad prognosis of this condition and avoid visual sequelae.
• Maxillofacial surgeons must be aware of this complication in a multidisciplinary context to adopt adequate treatment as soon as possible.

## 1 Introduction

Orbital cellulitis is a rare ophthalmic disease due to pansinusitis with hard maxillofacial management. It has fatal complications as cavernous sinus thrombosis, with a potentially poor functional prognosis. Early and appropriate treatment can improve the bad prognosis of this condition and avoid visual sequelae.

This article aims to highlight the importance of early diagnosis and adequate treatment of Cavernous Sinus Thrombosis. Therefore, without prompt medical management, the prognosis of Cavernous Sinus Thrombosis is always fatal.

## 2 CASE-REPOT

A 61-year-old female reported to our department by a regional referral hospital with right facial swelling, right proptosis, ophthalmoplegia, ptosis, decreased vision for 2 months, and normal vital signs parameters with no signs of sepsis detected at the time of the exam.

**Fig. 1:**
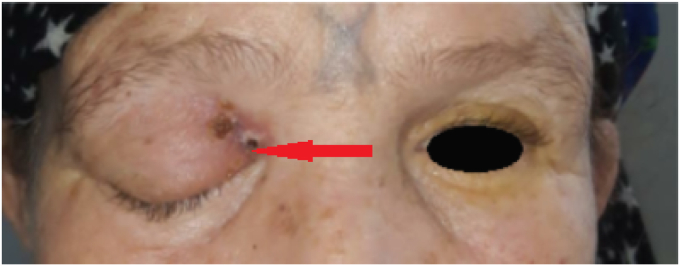
Patient with Orbital apex syndrome and cutaneous fistulised lesion.

She had a history of diabetes type 1 and she was receiving insulinotherapy and permanent headache; she had a fistulizing skin lesion at the upper palpebral level with a fever, ocular pain, and unknown sinusitis in the previous days. These signs had been present for two months, and started with facial orbital swelling (Fig. 1).

The patient was then hospitalized for orbital cellulitis. Biology found an inflammatory syndrome: WBC at 15,000 elt/mm3 - CRP at 200 mg/l and blood glucose at 3.2g/dl.

Without other concerns, an ophthalmologic evaluation was initially requested, which revealed a total loss of vision in the right eye with negative light perception. There are no abnormalities in her left eye.

**Fig. 2:**
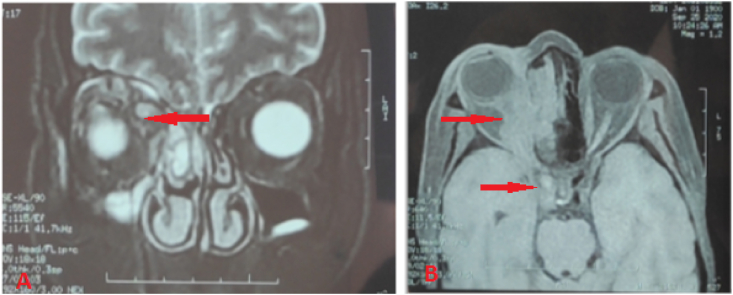
Magnetic resonance imaging (MRI) with improved contrast. (A) Coronal plane, periorbital swelling. (B) Distended superior ophthalmic vein on the right side, with a non-fatty density filling defect on the right side of the cavernous sinus.

**Fig. 3:**
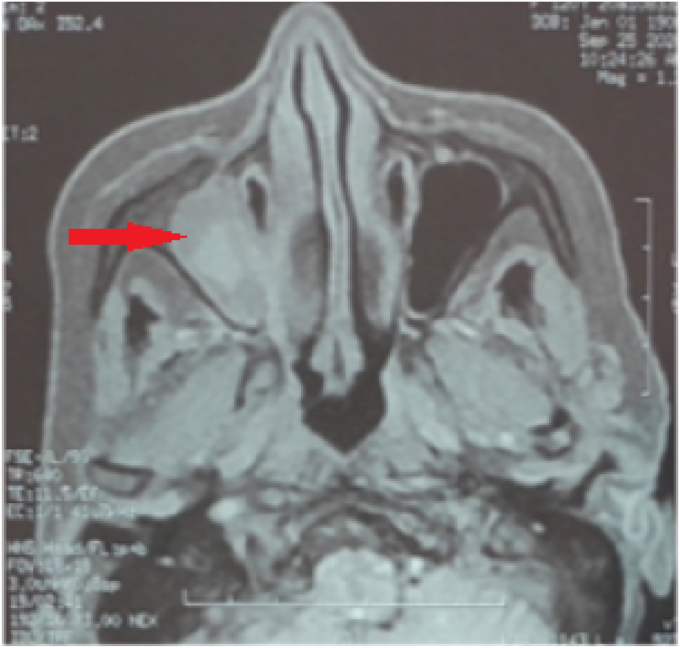
Filling of the right maxillary sinus.

MRI showed a dense and distended superior ophthalmic vein on the right side, with pre- and retro-septal swelling (chandler V). Computerized tomography confirmed the presence of a thrombus in her superior ophthalmic vein and cavernous sinus (Fig. 2, Fig. 3). The cranio-orbital MRI revealed sinusitis of the right maxilla.

In summary, Magnetic resonance imaging (MRI scan) showed an extension of superior ophthalmic vein thrombus to cavernous sinus thrombosis.

The patient was hospitalized for 5 days and she was then treated with a cycle of intravenous antibiotics (Amoxicillin-A clavulanic 1000mg/metronidazole 500mg), a heparin drip of 0.4 mg, and intravenous methylprednisolone 160 mg every 8 hours. The patient clinically responded to treatment with the recovery of the periorbital abscess (without the requirement of a drainage incision to evacuate the pus), but without resolution of the blindness already acquired due to the delay in diagnosis.

The patient was followed after her discharge on an external consultation by a rhythm of twice a month for two months and then referred for a follow-up in her home hospital centre. The patient improved clinically but still with permanent blindness.

This work has been reported in line with the SCARE 2020 criteria [13].

## 3 Discussion

Orbital cellulitis can be caused by a primary infection of the sinuses, skin, or teeth. Almost two-thirds of cases are attributable to a primary sinus infection, which is the most usual cause of orbital inflammation, and most are bacterial in origin [1]; 16% of cases are due to skin lesions like eczema, boils, or facial cellulitis [2]. Orbital cellulitis is the most common complication of acute sinusitis. The starting point is essentially ethmoidal, more rarely maxillary and frontal. It is a pathology that mainly affects teen people under fifteen years old [3] and adults between 60 and 70 years of age, with a preponderance of males.

Orbital cellulitis occurs in the three following conditions:

- Extension of an infection of the paranasal sinuses or other periorbital structures such as face, globe, or lacrimal sac
- Direct inoculation of the orbit after trauma, surgery, or cutaneous lesions
- Hematogenous propagation due to bacteremia

Immunosuppression, mainly diabetes, is a classic contributor in the dissemination of infection [4]. The clinical presentation combines inflammatory proptosis, palpebral edema, and reduced ocular movement (ophthalmoplegia).

Complications of orbital cellulitis may be limited to the orbit, such as subperiosteal or orbital abscess, neuropathic keratitis, or intracranial such as meningitis, sinus cavernous thrombosis, and cerebral abscess.

Cavernous sinus thrombosis in septic form occurs as a result of infection of the face, sinuses, or mouth. Very rarely, otitis media or the orbital sinuses are the cause. The documented incidence of cavernous sinus thrombosis as a complication of orbital cellulitis is 1%. Before the advent of antibiotics, the mortality due to sinus cavernous thrombosis was up to 100%, but it has been significantly decreased to 20–30% during the era of antibiotics [5].

Timely radiography imaging is essential in case of suspected orbital cellulite. An orbital CT scans with and without contrast injection or MRI scan are the key test for a positive diagnosis and sometimes etiological diagnosis [6]. It helps to determine the exact location and size of the orbital lesion and the state of the facial sinuses.

Our patient had a history of diabetes type 1, unknown maxillary sinusitis and had all the clinical features of orbital cellulitis. Imaging studies confirmed the diagnosis of sinus cavernous thrombosis. Radiological findings showed an expansion of the cavernous sinus.

Surgical treatment is required urgently if clinical signs worsen under treatment or if there is an optical neuropathy [7]. Orbital decompression surgery, drainage of an abscess, the opening of an infected sinus, or a combination of these procedures is advised in one of the listed circumstances:

- The vision is at risk.
- Suppuration or a foreign body is presumed.
- Imaging shows an orbital abscess or a large subperiosteal abscess, especially along the orbital roof.

Therapeutic management of the orbital complications of acute sinusitis is an emergency. It is always based on broad-spectrum antibiotic therapy. Surgery is justified in the collected forms [8]. Antibiotherapy is the cornerstone of the treatment. Most infectious agents are sensitive to penicillin, cephalosporin, and metronidazole [9]. The infection has improved thanks to antibiotics; a dual antibiotic therapy is used to eliminate the infection in our instance, the organism was sensitive to penicillin and metronidazole. Prolonged treatment and extensive debridement are recommended [1].

An orbital cellulitis is a serious infectious condition of the orbit. It entails a high potential risk of various complications: optic neuritis, blindness, meningitis, cerebral abscess, intracranial empyema, cavernous sinus thrombosis, and even death [10].

Cavernous sinus thrombosis is a rare but highly fatal complication of orbital cellulitis, and it is related to thrombophlebitis in the ophthalmic vein. Valveless upper and lower ophthalmic veins are the first access point for the extension of infection within the facial sinuses to the cavernous sinus [11]. This complication may also be a consequence of a systemic infection that can lead to secondary inflammatory and procoagulant responses [12].

## 4 Conclusion

Even if it is of very low incidence, it can manifest itself. Therefore a rapid diagnosis, radiography imaging in time, a multidisciplinary perspective and appropriate therapeutic management are the cornerstones of reducing morbidities, such as blindness due to septicemia, and death caused by a sinus thrombosis cavernous complication.

## Provenance and peer review

Not commissioned, externally peer reviewed.

## Declaration of competing interest

Authors of this article have no conflict or competing interests. All of the authors approved the final version of the manuscript.

## Annals of medicine and surgery

The following information is required for submission. Please note that failure to respond to these questions/statements will mean your submission will be returned. If you have nothing to declare in any of these categories then this should be stated.

## Registration of research studies

In accordance with the Declaration of Helsinki 2013, all research involving human participants has to be registered in a publicly accessible database. Please enter the name of the registry and the unique identifying number (UIN) of your study.

You can register any type of research at http://www.researchregistry.com to obtain your UIN if you have not already registered. This is mandatory for human studies only. Trials and certain observational research can also be registered elsewhere such as: ClinicalTrials.gov or ISRCTN or numerous other registries.

1. Name of the registry:
2. Unique Identifying number or registration ID: 6313
3. Hyperlink to your specific registration (must be publicly accessible and will be checked):
